# Development and comparison of immunologic assays to detect primary RSV infections in infants

**DOI:** 10.3389/fimmu.2023.1332772

**Published:** 2024-01-12

**Authors:** Larry J. Anderson, Samadhan J. Jadhao, Laila Hussaini, Binh Ha, Courtney E. McCracken, Theda Gibson, Inci Yildirim, Jumi Yi, Kathy Stephens, Chelsea Korski, Carol Kao, Heying Sun, Chun Yi Lee, Anna Jaunarajs, Christina A. Rostad, Evan J. Anderson

**Affiliations:** ^1^ Division of Infectious Diseases, Department of Pediatrics, Emory University School of Medicine, Atlanta, GA, United States; ^2^ Center for Childhood Infections and Vaccines, Children’s Healthcare of Atlanta, Atlanta, GA, United States; ^3^ Department of Pediatrics, Emory University School of Medicine and Children’s Healthcare of Atlanta, Atlanta, GA, United States; ^4^ The Emmes Company, Rockville, MD, United States; ^5^ Division of Infectious Diseases, Department of Medicine, Emory University School of Medicine, Atlanta, GA, United States

**Keywords:** respiratory syncytial virus, diagnosis, serology, infant, infection

## Abstract

Effective respiratory syncytial virus (RSV) vaccines have been developed and licensed for elderly adults and pregnant women but not yet for infants and young children. The RSV immune state of the young child, i.e., previously RSV infected or not, is important to the conduct and interpretation of epidemiology studies and vaccine clinical trials. To address the need for sensitive assays to detect immunologic evidence of past infection, we developed, characterized, and evaluated 7 assays including 4 IgG antibody enzyme immunoassays (EIAs), two neutralizing antibody assays, and an IFN-γ EliSpot (EliSpot) assay. The four IgG EIAs used a subgroup A plus subgroup B RSV-infected Hep-2 cell lysate antigen (Lysate), an expressed RSV F protein antigen (F), an expressed subgroup A G protein antigen (Ga), or an expressed subgroup B G protein (Gb) antigen. The two neutralizing antibody assays used either a subgroup A or a subgroup B RSV strain. The EliSpot assay used a sucrose cushion purified combination of subgroup A and subgroup B infected cell lysate. All seven assays had acceptable repeatability, signal against control antigen, lower limit of detection, and, for the antibody assays, effect of red cell lysis, lipemia and anticoagulation of sample on results. In 44 sera collected from children >6 months after an RSV positive illness, the lysate, F, Ga and Gb IgG EIAs, and the subgroup A and B neutralizing antibody assays, and the EliSpot assays were positive in 100%, 100%, 86%, 95%, 43%, and 57%, respectively. The Lysate and F EIAs were most sensitive for detecting RSV antibody in young children with a documented RSV infection. Unexpectedly, the EliSpot assay was positive in 9/15 (60%) of PBMC specimens from infants not exposed to an RSV season, possibly from maternal microchimerism. The Lysate and F EIAs provide good options to reliably detect RSV antibodies in young children for epidemiologic studies and vaccine trials.

## Introduction

Human respiratory syncytial virus (RSV) is a leading cause of serious respiratory tract infection in young children worldwide (1–4). Primary RSV infection often occurs during an infant’s first seasonal RSV exposure, and nearly all children are infected by 2 years of age (5–12). In addition to acute illness, RSV infection in infancy has been linked to later respiratory morbidity and the development of asthma (13–15). Since RSV infection induces incomplete protective immunity, repeat RSV infections and disease occur throughout life, with an especially severe disease in those with compromised cardiac, pulmonary, or immune systems and in the elderly (16).

The burden of RSV disease has made it a high priority for vaccine development. After over 60 years of research, the first vaccines, two for elderly adults and a maternal vaccine to protect the young infant, have been licensed (17–20). The maternal vaccine and a long-acting monoclonal antibody are designed to protect the young infant, but a vaccine for administration to the young child is not yet available. Developing a vaccine for this target population continues to be challenging with one barrier being the concern—that the enhanced RSV disease (ERD) associated with an earlier administration of the first RSV vaccine, a formalin-inactivated, tissue culture grown RSV with alum adjuvant that was studied in the 1960s (21–24), might occur with other non-live virus vaccines in young, RSV naive children. This concern has limited vaccine development for the young child to live attenuated RSV or RSV proteins expressed in a virus vector or by mRNA. Achieving the right balance between safety and immunogenicity and efficacy for a vaccine for this target population remains elusive. The clinical trials needed to evaluate vaccines and identify one that is safe and effective for the young child will benefit from understanding the pre-vaccination RSV immune state of the child, i.e., previously RSV-infected or not. Antibody assays are usually used to determine if a young child has been previously infected, but a young child’s infection may be missed due to a poor initial response to infection and/or due to waning immunity (9, 25–31). RSV antibodies have been detected by a variety of assays including functional assays such as neutralization, fusion inhibition, and Fc-induced activity, binding enzyme immunoassays (EIA) to detect IgG, IgA, and/or IgM antibodies against a variety of RSV proteins, and blocking assays to detect epitope- or antigenic-site-specific antibodies (11, 27, 31–46). Since the optimal assay or combination of assays to detect a past infection in the young child has not been determined, we chose to address this gap by developing, characterizing (assay repeatability, reactivity against control antigen or specimen, lower limit of detection, and the effect of specimen quality or processing on results), and evaluating the six antibody assays and one T cell assay for their ability to detect evidence of RSV immunity in young children. The antibody assays included two neutralizing antibody assays (one each for RSV subgroups A and B strains) and four binding antibody EIAs (i.e., one with subgroup A and B lysate antigen (Lysate), one with expressed F protein antigen (F), one with expressed subgroup A G protein antigen (Ga), and one with expressed subgroup B G protein antigen (Gb)). We chose the F and G protein EIAs because these proteins are the only ones that induce neutralizing antibodies and high levels of protection in animal studies (47, 48). The lysate EIA was used because it contains multiple proteins produced by RSV such as the F, G, N, M, and P proteins (26, 49). We included the neutralizing antibody assays because neutralizing antibodies are correlated with protection from disease. We included the INF-γ EliSpot (EliSpot) assay because we thought that T cell responses might detect some past infections missed by the antibody assays and would not be affected by the maternal immunity transferred to the fetus. After characterizing the assays, we compared the ability of these seven assays to detect evidence of infection-induced or maternal-acquired RSV immunity in four groups of blood specimens with different types of RSV exposures. The overall goal of these studies was to determine which assay or combination of assays would be most sensitive for detecting evidence of infection-induced or maternal-acquired RSV immunity.

## Materials and methods

### Patients and specimens

We enrolled four groups of patients under an Emory IRB-approved protocol between July 2015 and September 2018. The groups were chosen to address different aspects of assay performance. Specimens and patient-associated data were collected after informed consent was obtained. The CDC definition was used to determine the RSV season, i.e., onset is the first of two consecutive weeks with ≥10% of RSV tests being positive and the end as the last of two consecutive weeks with >10% of the detections being positive (50).

#### Group A (specificity of the INF-γ EliSpot assay)

Cord blood was collected at the time of delivery if delivery occurred >4 months after the last RSV season and before the next RSV season at Emory University Hospital Midtown, Atlanta, GA, USA. After maternal consent has been obtained, cord blood was collected using cord blood collection kits with citrate phosphate dextrose (CPD) solution (Pall Corporation, Port Washington, NY, USA) and transferred to the laboratory where it was processed for plasma and cord blood mononuclear cells (CBMCs). Once separated, the plasma and CBMCs were divided into aliquots, and the plasma was stored at -80°C and the CBMCs in liquid nitrogen for later testing. A dilution factor was used to account for the CPD dextrose solution in the collection kit and solutions used in the separation of plasma from CBMCs.

#### Group B (specificity of the INF-γ EliSpot assay)

Healthy, RSV unexposed infants, i.e., infants >4 months old who were born after the previous year’s RSV season ended, were enrolled; they were children seen in the Egleston Hospital Emergency Department (ED) or Hughes Spalding Hospital Primary Care or admitted to Egleston Hospital in Atlanta, GA, USA before the next RSV season. After parental consent was obtained, blood specimens were collected and clinical and demographic data were retrieved from the clinical record. Blood (volume appropriate for age and weight of the infant) was collected in buffered sodium citrate cell preparation tubes (CPT) and transported to the laboratory for processing. The peripheral blood mononuclear cells (PBMCs) were separated from the plasma, and each sample was divided into aliquots and stored at -80°C for the plasma and in liquid nitrogen for the PBMCs.

#### Group C (assay sensitivity)

Otherwise healthy children exposed to their first RSV season who were seen in the Egleston Hospital ED or admitted to Egleston Hospital or Scottish Rite Hospital in Atlanta, GA, USA with an RSV-positive illness and whose parents consented were enrolled and asked to return ≥6 months later and before the start of the next RSV season to have a blood specimen collected. RSV positivity was determined in physician-ordered specimens by a BioFire respiratory panel PCR (Salt Lake City, UT, USA). Those who returned had a blood specimen collected and their clinical and demographic profile retrieved from the clinical record. The blood specimen (volume appropriate for age and weight of the infant) was collected in buffered sodium citrate CPT tubes and transported to the laboratory for processing. The PBMCs were separated from the plasma, and each was divided into aliquots and stored at -80°C for the plasma and in liquid nitrogen for the PBMCs.

#### Group D (performance of selected assays in blood specimens from children with unknown RSV infection status)

Otherwise healthy children who were seen in the Egleston Hospital ED or Hughes Spalding Hospital Primary Care or admitted to Egleston Hospital in Atlanta, GA, USA >4 months after the end of their first RSV season and before the start of the next RSV season were enrolled. After parental consent was obtained, clinical and demographic data were collected from the clinical record and blood (volume appropriate for age and weight of the infant) was collected in buffered sodium citrate CPT tubes (Beckton Dickinson, Franklin Lakes, NJ, USA) and transported to the laboratory where it was processed. The PBMCs were separated from the plasma, and each was divided into aliquots and stored at -80°C for the plasma and in liquid nitrogen for the PBMCs.

### Antigens and virus

#### Lysate antigen

The antigen for the lysate EIA is lysate from A2 (ATCC catalogue number VR 1540), a subgroup A RSV strain, and B1 strain (ATCC catalogue number VR 1400), a subgroup B RSV strain, infected cells (51). The viruses were grown in HEp-2 cell (ATCC^®^ CCL23™ HEp-2 cells) under MEM with 5% fetal bovine serum (FBS) at a multiplicity of infection (MOI) of 0.1 TCID_50_ per cell. After 2 days, the media was changed to serum-free medium to minimize non-RSV antigens in the lysate. At cytopathic effect (CPE) of 3+ to 4+ or day 3 or 4 post-inoculation, the cells were harvested by scraping from the flask, and cells and media were centrifuged at 3,000 ×*g*. The supernatant was transferred to 50-mL tubes, and the cell pellets were pooled, lysed with a 1/10 dilution of lysis buffer (Cell Signaling Technology, MA, USA) on ice, and sonicated. The treated cell pellet was combined with supernatants, frozen and thawed three times, and clarified by centrifugation at 3,000 ×*g* for 30 min. Halt Protease inhibitor (Thermo Scientific, USA) was added to the clarified supernatants which were divided into aliquots (A2 and B1 separately) and stored at -80°C.

#### F, Ga, and Gb antigens

The antigens for the F, Ga, and Gb EIAs were produced by expressing a human codon-optimized secreted portion of RSV, not prefusion-stabilized F (A2 strain), Ga (A2 strain), or Gb (B1 strain) gene with sequences encoding 6X histidine tag at the carboxyl terminus as previously described (51). Briefly, the respective genes were cloned into inducible plasmid pCDNA5/TO vectors with a secretory signal to release the expressed protein into the medium and woodchuck hepatitis virus post-transcriptional regulatory element to increase the expression. The cloned F, Ga, or Gb pCDNA5/TO vector plasmid constructs were transfected into the 293F/Bla cell line under antibiotic selection to develop stably transfected cell lines. Production of the appropriate protein from the stably transfected cells was confirmed by Western blot with the human monoclonal antibody (mAb) Motavizumab (MedImmune, LLC, Gaithersburg, MD, USA) to detect F and the human mAb 3D3 (kindly provided by Trellis Bioscience, LLC, Redwood City, CA, USA) to detect Ga and Gb. The histidine tag on the F, Ga, and Gb proteins was detected with an anti-histidine mAb (cat no. OB05, Sigma Inc., USA). The stably transfected cells were stored in liquid nitrogen.

#### Antigen titration

The titer of antigen for the EIAs was determined by adsorbing serial twofold dilutions in carbonate buffer (pH 9.6) onto EIA plates (Maxisorb, Nunc, USA) and detecting the lysate, F, Ga, Gb, or control antigens with a goat anti-RSV antibody (MilliporeSigma, Burlington, MA, USA) or a high-titer human anti-RSV antibody (catalog number BEI NR-4021, BEI Resources, Manassas, VA, USA) followed by peroxidase-conjugated donkey anti-goat, or anti-human, IgG antibody (Jackson Immuno Research Labs, West Grove, PA, USA), developing color with OPD/peroxide solution, and reading the absorbance at 490 nm. The highest antigen dilution that maintained a high P–N absorbance signal (P = absorbance for the RSV antigen and N = absorbance for the control antigen) was used to coat the plates for the antibody EIAs.

#### Virus for neutralization assays

Subgroup A (A2 strain of RSV, ATCC catalog # VR 1540) and subgroup B [RSV B1, B WV/14617/85;173: 829-839, ATCC catalog # VR 1400 (52)] viruses were individually grown in ATCC CCL23 HEp-2 cells at m.o.i. of 0.01 in 175-cm^2^ flasks. At 4+ cytopathic effect (CPE), the media were adjusted to 25% sucrose and the flasks frozen at -80°C. After thawing, the virus containing medium in the flasks was pooled, clarified by centrifugation at 1,000 rpm (208 ×*g*) in a refrigerated Model 5810 R Eppendorf Centrifuge, divided into aliquots, and stored at -80°C.

#### RSV for EliSpot assay

Sucrose gradient-purified subgroup A (RSV A2, ATCC catalog # VR 1540) plus subgroup B (RSV B1, ATCC catalog # VR 1400) was used as antigen for the EliSpot assay. The viruses were individually grown in ATCC CCL23 HEp-2 cells at 0.01 m.o.i. in 175-cm^2^ flasks to 4+ cytopathic effect (CPE), and the flasks were frozen at -80°C. After thawing, the virus-containing medium in the flasks was pooled and clarified by centrifugation at 600 ×*g* for 10 min in a refrigerated Eppendorf Centrifuge (Model 5810 R). The clarified virus-containing supernatant was transferred to plastic ultra-centrifugation tubes to which an underlay of 4 mL of sterile 20% sucrose in PBS was added, and the tubes were centrifuged for 2 h at 10,000 or 17,000 RPM in a SW32 Ti rotor, Beckman Coulter Optima-L-90K Ultracentrifuge at 4°C. The supernatant and sucrose cushion were aspirated without touching the virus-containing pellet. The pellet was re-suspended in 3 mL of cold MEM without FBS or BSA, divided into aliquots, fast-frozen, and stored at -80°C. The infectivity titer of the stock of an aliquot of purified virus was determined after storage at -80°C for >24 h as described below. The infectivity titer for sucrose-purified RSV A2 should be >10^6.0^ TCID_50_/mL and for RSV B1 >10^4.5^ TCID_50_/mL.

#### Infectivity titration

The titer of infectious virus was determined with serial 10-fold dilutions of the virus (10^-1^ through 10^-8^ dilutions) in HEp-2 cells in 96-well micro-titer plates. Virus replication was detected after 4 days by an RSV antigen EIA by fixing the cells with paraformaldehyde followed by washing the plates two times with PBS, blocking with blocking buffer (0.33% fish skin gelatin, 0.33% skim milk, and 0.33% bovine casein (GMC buffer)) for 2 h at 37°C, washing with PBS plus 0.5%Tween (PBS-T), adding goat anti-RSV antibody (MilliporeSigma, Burlington, MA, USA) in GMC buffer plus 0.15% Tween 20 (GMC-T) for 1 h, washing with PBS-T, adding peroxidase-conjugated donkey anti-goat IgG antibody (Jackson Immuno Research Labs, West Grove, PA, USA) in GMC-T, and adding OPD/H_2_O_2_ to develop color. Absorbance was read at 490 nm. Wells with absorbance >mean + three standard deviations of absorbance for uninfected cells were considered positive for virus replication. The viral infectivity titer (TCID_50_/mL on HEp-2 cells) was calculated by using the Spearman–Karber method (53). One hundred TCID_50_ was added to each well for the neutralization assays. Furthermore, 1/10 of virus A2 plus 1/10 of virus B1 by volume was used for the EliSpot assay.

### Assays

#### EIA antibody assays

The lysate, F protein, Ga protein, and Gb protein IgG antibody EIAs were performed as previously described (51). Briefly, the respective antigen diluted in sodium carbonate bicarbonate coating buffer (pH 9.6) was adsorbed onto the EIA plates for 2 h at 37°C followed by overnight at 4°C. The antigen-coated plates were washed two times with PBS, blocked with blocking GMC buffer for 2 h at 37°C, and washed with PBS-T before adding the serum or plasma, i.e., the reference standard sera or patient plasma, diluted 1/200 in GMC buffer plus 0.15% Tween 20 (GMC-T). Higher dilutions of patient specimens were used for specimens with a high titer of antibody that had absorbance readings that were too high to estimate the titer. All specimens were tested in three wells with the respective RSV antigen and three wells with the corresponding control antigen. A run (four to six plates/run) reference standard curve was generated with twofold serial dilutions of the BEI NR 4021 serum beginning at 1:100 dilution for Ga and Gb EIAs and 1:200 dilution for F and lysate EIAs. In addition, each plate in the run had reference standard sera diluted to give low (BEI NR 4023 serum diluted to give absorbance ~200) and medium (BEI NR 4022 serum diluted to give absorbance between 500 and 1,000) positive absorbance readings and a negative control (IgG-depleted) serum. The specimens, reference sera, and control sera were incubated for 110 min (± 10 min) at 37°C, the plates were then washed with PBS-Tween (0.05%), horseradish peroxidase-conjugated donkey anti-human IgG antibody diluted in GMC-T was added and incubated for 70 min ( ± 5 min) at 37°C, the plates were washed with PBS-T, and color was developed with the addition of OPD/H_2_O_2_ solution for 30 min. At 30 min (± 3 min), the reaction was stopped with 4 N H_2_SO_4_, and absorbance was read at 490 nm. A specimen was considered positive if the mean absorbance of the RSV antigen wells (P) was greater than the mean absorbance plus three SD of control antigen wells (N), the P-N value was greater than the mean of the no-antibody control wells, and the mean P-N value was greater than the mean P-N value for IgG-depleted serum. We estimated the antibody titer by referring the P-N value to a four-parameter logistic regression model (4PL) based on the reference standard curve. The 4PL model was generated with SAS version 9.4 (Cary, NC, US). The data were reported as the estimated antibody titer of the specimen.

#### Neutralizing antibody assays

For both subgroup A and B neutralizing antibody assays, heat-inactivated serum or plasma was serially diluted in MEM/0.5% FBS with penicillin and streptomycin in sterile U-bottom microtiter plates followed by the addition of 100 TCID_50_ of virus and incubation for 1 h ± 5 min at room temperature. The virus-sera/plasma mixture was then added to previously seeded Hep-2 cells at 50% to 70% confluence and incubated at 37°C under 5% CO_2_ for 3 days for A2 virus neutralization and 4 days for B virus neutralization. Each run includes one plate with twofold serial dilutions (1:200-1/titration of the BEI high RSV antibody titer serum; BEI NR 4021) and a back-titration of the virus inoculum (serial fourfold dilutions of the virus inoculum from undiluted to a 1/1024 dilution). After 3 or 4 days of incubation, the media was aspirated and the cells fixed with 50 μL of freshly prepared 4% paraformaldehyde/PBS solution for 15 min at room temperature, washed two times with PBS, and incubated with 0.3 M glycine Tris solution (pH 7.4) for 30 min; after aspiration of the glycine solution, the plates were tested for RSV replication by EIA with a goat anti-RSV antibody (MilliporeSigma, Burlington, MA, USA) followed by peroxidase-conjugated donkey anti-goat IgG antibody (Jackson Immuno Research Labs, West Grove, PA, USA), developing color with OPD/peroxide solution and reading the absorbance at 490 nm. Wells with P-N (P is the absorbance reading for the well and N is the mean of wells with no virus added) greater than (P-N)/2 (P is the mean of wells with virus inoculum and no antibody and N is the mean of wells with no virus added) were considered positive for neutralization. The neutralizing antibody titer was calculated by using the Spearman–Karber method (53) and reported as such.

#### RSV INF-γ EliSpot assay

On day 1 of the assay, PBMCs were prepared by thawing and resting overnight at 37°C in a 5% CO_2_ incubator in HR10 media (RPMI media; Mediatech, Manassas, VA, USA), heat-inactivated human AB serum, penicillin/streptomycin (Mediatech), glutamax (Gibco, St. Louis, MO, USA), MEM non-essential amino acids (Gibco), and sodium pyruvate (Mediatech). On day 1, ELISpot MSIPS plates (Millipore) were also prepared by coating the plates with IFN-γ capture monoclonal antibody 1-D1K (Mabtech AB, Nacka Strand, Sweden), wrapping the plates in parafilm and incubating the plates overnight for at least 16 h at 2–8°C. On day 2, the anti-human IFN-γ mAb 1-D1K was removed and 200 μL of HR10 EliSpot cell culture media was added for 2 h at 37°C as blocking solution followed by the addition of 2.0 × 10^5^ live PBMCs plus the RSV antigen, control antigen, or PHA per well and incubating the plate at 37°C under 5% CO_2_ for 40 h. On day 4, after the 40-h incubation, the PBMCs were removed, the plate was washed with an automatic plate washer, and biotinylated anti-INF-γ detection antibody 7-B6-B6 (Mabtech) was added and incubated for 2 h at room temperature. The plates were then washed again and streptavidin–horse radish peroxidase (HRP) conjugate (BD Biosciences, San Jose, CA, USA) was added and incubated for 1 h; after washing the plates, AEC Substrate Kit (BD Biosciences, San Jose, CA, USA) was added for 5 min, the plate was rinsed with cold water and dried overnight, and color spots were read with S6 FluoroCore ImmunoSpot (Cellular Technology Limited, Shaker Heights, OH, USA) EliSpot reader. The number of spots was reported as spots/10^6^ cells (spots detected for 2 × 10^5^ cells multiplied by 5). Specimens with the average number of spots for the RSV antigen wells >25 spots/10^6^ cells (number of spots > mean + two standard deviations for negative control wells for all tested specimens) and > mean + three SD of the control antigen wells for a given specimen were considered positive RSV responses.

### Statistical analysis

Titers for each antibody assay and number of spots per 10^6^ cells for the EliSpot assay were summarized descriptively using fraction and percent positive, geometric mean, 95% confidence interval, standard deviation, and median.

Comparisons of the rate of positivity in each assay to the lysate EIA assay were performed using *p*-values obtained from Fisher’s exact test. The false discovery rate was controlled for by using the Benjamini–Hochberg procedure. A comparison of the rate of EliSpot assay positivity between subjects in groups B and C was also performed using Fisher’s exact test.

To assess maternal antibody half-life, a line of best fit was obtained from a plot of lysate EIA antibody titers *versus* age in days. One plot was generated for group B (unexposed) subjects alone and one was generated for group A (maternal) and group B subjects combined, where the age of 0 days was used for all group A subjects. There was no overlap in the subjects belonging to group A and group B, and each subject only had one lysate EIA assay titer. Half-life estimates were obtained using the following equation:


$$t_{1/2}=\ln(2)/k_{el}$$

where *k*_el_ is the first-order elimination rate constant given by the slope of the line of best fit.

## Results

### Patients

With the exception of group C, results were available for analysis from most subjects (**Table 1**). In group A, 60 of 62 enrolled pregnant women had an adequate specimen collected and 59 of these 60 had a response to the PHA-positive control indicating the presence of functional T cells for the INF-γ EliSpot. In group B, 39 of 39 enrolled children had an adequate specimen collected and acceptable test results. In group C, 102 of 220 enrolled children returned for blood collection and had an adequate specimen collected at ≥6 months after enrollment, which approximates the time of their RSV positive infection. The reasons for non-return are given in **Table 1**. In group D, 124 of 126 enrolled children had an adequate specimen collected.

**Table 1:** Enrollment.

Subject disposition and characteristics	Group A Cord blood	Group B RSV unexposed	Group C RSV positive	Group D RSV exposed
Screened	67	44	220	138
Enrolled (percent of screened subjects)	62 (93%)	39 (89%)	220 (100%)	126 (91%)
Study subjects (percent of enrolled subjects)^a^	59 (95%)	39 (100%)	102 (46%)	124 (98%)
Median age (range)	30 years (20–42)	138 days (121–203)	86^b^ days (16–238)	390 days (270–557)
Female	59	21	39	51
Asian	2	0	3	1
Black or African American	51	31	33	90
Multiple including American Indian, Alaska Native, Asian, Black or African American, Native Hawaiian or other Pacific Islander, and White	0	3	8	10
White	6	5	56	25

a Subjects who were enrolled and had specimens collected and test results available. For group C, subjects were enrolled at the time they had a RSV PCR+ illness but had to return ≥ 6 months to have a blood specimen collected to be included in the study. Among the 118 children not included, 78 were lost to follow-up, 29 had protocol deviations, were ineligible at or after enrollment, or returned after the RSV season, and 11 were withdrawn by their parent or guardian.

b Age at enrollment which is ~age at infection. The blood was drawn ≥ 6 months after enrollment. The median age at blood draw was 302 days, with a range of 203–558 days.

### Characteristics of lysate, F, Ga, and Gb antigens in antibody EIAs

As previously described, the expressed F, Ga, and Gb proteins gave the appropriately sized bands by Western blot (51). The respective protein-specific and lysate antibody EIAs detected a high titer of RSV antibody in the BEI NR 4021 high-RSV antibody titer serum and had acceptable reactivity against two negative controls, control antigen, and IgG-depleted serum (**Supplementary Tables S1**, **S2**). The antibody titer was minimally affected by red cell lysis (blood specimen stored overnight at 4°C), lipemia (blood collected within 1 h of eating), and anticoagulation (blood collected in sodium citrate CPT tubes) when compared to a fasting serum specimen in five adult blood specimens (**Supplementary Table S3**). The blood specimens from a given adult were collected on the same day under an IRB-approved blood collection protocol and delinked from personal identifying information. The ratio for the specimen type, i.e., with red cell lysis, lipemia, or anticoagulation, antibody titer over the corresponding reference specimen titer for each study subject and assay, was used to compare the effects among specimen types. We chose the fasting serum specimen titer to be our reference for this ratio. The median ratio for the four EIAs for the five adult specimens was 0.94 (6% decrease with a range of 1%–6%) for lipemia, 0.88 (12% decrease with a range of 2%–17%) for hemolysis, and 0.87 (13% decrease with a range of 0% to 13%) for anticoagulation. The EIAs detected high titers of antibody with good consistency. The lysate and F EIAs detected high median antibody titers for the BEI NR 4021 reference sera of 253,650 and 328,663, respectively (**Supplementary Table S1**). The Ga and Gb EIAs detected a lower median titer of antibodies—54,074 and 71,728, respectively. We used the coefficient of variation (CV), i.e., standard deviation/mean, to evaluate assay repeatability. We considered a CV <30%, which gives a mean + three standard deviations less than <2-fold different from the mean, as acceptable. All four EIAs had acceptable repeatability as indicated by CVs of titers for six replicates of 12 adult serum specimens between 0.07 and 0.22 (**Table 2**). The titer of antibody that had a >95% chance of a positive result in the four EIA assays was ~300.

**Table 2:** Repeatability of the assays (the table summarizes the specimens tested for assay repeatability based on the coefficient of variation).

Assay	No. Spec	Repetitions/specimen^a^	Days^b^	Median specimen titer or spots/10^6^ cells	Range of specimen titers or spots/10^6^ cells	Median of specimen CVs	Range of specimen CVs
A Neut	7	4	2	158	138–846	0.25	0.12–0.53
B Neut	7	4	2	192	24–2,345	0.26	0.0–0.41
Lysate EIA	12	6	2	40,567	4,356–171,436	0.22	0.16–0.31
F EIA	12	6	2	33,149	4,296–329,318	0.07	0.01–0.21
Ga EIA	12	6	2	4,516	825–24,241	0.08	0.01–0.22
Gb EIA	12	6	2	6,948	584–33,668	0.1	0.01–0.25
INF-γ EliSpot	5	3–6	2–3	279^c^	81–1043^c^	0.18	0.04–0.26

A Neut, neutralizing antibodies against the subgroup A RSV strain A2; B Neut, neutralizing antibodies against the subgroup B RSV strain B1; Lysate EIA, IgG antibody enzyme immunoassay (EIA) with lysate from A2 and B1 infected Hep-2 cells antigen; F EIA, IgG antibody against expressed F protein antigen; Ga EIA, IgG antibody against expressed A2 strain G protein as antigen; Gb EIA, IgG antibody against expressed B1 strain G protein antigen; INF-γ EliSpot, uses sucrose cushion-purified RSV A2 and B1 from Hep-2 cells for stimulation; No. Spec, number of sera, plasma, or PBMC specimens tested; CV, coefficient of variation for the replicate tests for each specimen.

a Replicates = total number of replicates for each specimen.

b Number of separate days specimens were tested.

c Spots/10^6^ cells.

#### Neutralizing antibody assays

The subgroup A and B neutralizing antibody assays gave similar titers against the high RSV titer BEI NR 4021 high-RSV antibody titer serum of 1,810 and 1,290, respectively (**Table 2**). Lipemia and anticoagulation had minimal effect on subgroup A neutralizing antibody titers relative to fasting serum as indicated by the median decrease in antibody titer for the five adult specimens of 0% and 6%, respectively. There was a greater effect of hemolysis with a median decrease in antibody titer of 67%. The repeatability was acceptable as indicated by the median CV for four replicates of seven adults’ serum specimens of 0.25 for the subgroup A neutralization assay and 0.26 for the subgroup B assay. The range of CVs for the two assays was 0.12–0.53 and 0.0–0.41, respectively.

#### EliSpot INF-γ assay

The EliSpot assay detected responses, as expected, in adult PBMCs with an average of 279 spots/10^6^ cells and a range of 81–1,043. The repeatability of replicate testing was acceptable with a median CV of 0.18 and range of 0.04–0.26 (**Table 2**). There was a good response to the PHA-positive control stimulation with a median of 1,978 spots/10^6^ cells and range of 420–4,000. A positive response to the RSV antigen of 125 spots/10^6^ cells was detected in one of 59 negative control CBMC specimens.

#### Assay comparison

We compared the seven assays in a subset of randomly selected specimens from 44 children from group C (children with an RSV-positive illness) and from 15 children from group D (children not exposed to an RSV season). This subset testing was an efficient way to determine which assay or assays should be used to test all specimens. The results for specimens from group C indicated that the lysate and the F EIAs were the most sensitive assays. Both assays detected RSV antibodies in 44/44 (100%) of these plasma specimens (**Table 3**). In comparison, the Ga EIA was positive in only 19/44 (43%) and the Gb EIA in 25/44 (57%) of these specimens. In 36/44 (82%) of the specimens, G protein antibodies were detected, i.e., either subgroup A or subgroup B or both. Subgroup A neutralizing antibodies were detected in 38/44 (86%) and subgroup B neutralizing antibodies in 42/44 (95%) and either subgroup A or B or both neutralizing antibodies in 42/44 (95%) of these group C specimens. With the EliSpot, 38/44 (86%) of the group C PBMCs were positive. The difference in positivity was significant (*p* < 0.05) between either the lysate or F EIA and the other assays, with the exception of the subgroup B neutralizing antibody assay (**Supplementary Table S4**). As expected, most (between 60% for the Ga EIA and 100% for the lysate and F EIAs) of the 15 specimens from group D (children not exposed to an RSV season) were antibody-positive since all would have had residual maternal antibody (**Table 3**). Unexpectedly, the EliSpot was positive in 60% of the 15 group B PBMC specimens but with a lower median of 58 spots/10^6^ cells (range, 40–113) than the group C specimens which had a median of 138 spots/10^6^ cells (range of 30–1,030). This finding suggests that the EliSpot assay is not specific for detecting infection-induced immunity in young children. Overall, these results indicated that the lysate and F EIAs are the most sensitive assays for detecting a prior infection, and consequently, we focused on these two assays for the remaining studies.

**Table 3:** Comparison of assay sensitivity in a subset of group C and D specimens.

Assay	Group	Fraction positive *n*/*N*	Percent positive (%)	GMT (95% CI)	STD	Median^a^	Min, max
A Neut	C	38/44	86	24.9 (18.3, 33.8)	44.7	26	0, 160
	B	14/15	93	20.6 (14.1, 30.2)	14.6	28	0, 57
B Neut	C	42/44	95	47 (34.1, 64.9)	60.8	57	0, 160
	B	14/15	93	18.7 (12.2, 28.8)	37.8	18	0, 160
Lysate EIA	C	44/44	100	4,808.5 (3,450.8, 6,700.4)	8244.4	5,882	415, 39,584
	B	15/15	100	1,573.7 (1,026.1, 2,413.8)	2572.4	1,468	537, 10,970
F EIA	C	44/44	100	5,652.6 (3,697.6, 8,641.2)	14842.9	8,313	327, 74,424
	B	15/15	100	2,188.3 (1,438.9, 3,327.9)	4700.5	2,066	998, 19,884
Ga EIA	C	19/44	43	999.2 (619.0, 1,612.9)	1274.1	0	0, 5,619
	B	9/15	60	604.9 (373.2, 980.5)	514.2	331	0, 1,536
Gb EIA	C	25/44	57	2,379.2 (1,427.0, 3,966.9)	6,944.4	647	0, 41,112
	B	11/15	73	907.4 (513.9, 1,602.2)	1,093.0	455	0, 3,528
INF-γ EliSpot	C	38/44	86	118.1 (81.9, 170.4)	250.1	112	<25^b^, 1,030
	B	9/15	60	38.3 (26.5, 55.3)	30.6	40	<25, 113

The results are from a subset of 44 randomly selected specimens from group C (PCR-positive RSV infections) and 15 randomly selected specimens from group B (not exposed to an RSV season). Note that 44 of the 45 group C specimens were selected. One of the 45 did not have an adequate EliSpot test and was excluded from this analysis.

A Neut, neutralizing antibodies against the subgroup A RSV strain A2; B Neut, neutralizing antibodies against the subgroup B RSV strain B1; Lysate EIA, IgG antibody enzyme immunoassay (EIA) with lysate from A2 and B1 infected Hep-2 cells antigen; F EIA, IgG antibody against expressed F protein antigen; Ga EIA, IgG antibody against expressed A2 strain G protein as antigen; Gb EIA, IgG antibody against expressed B1 strain G protein antigen; INF-γ EliSpot, uses sucrose cushion-purified RSV A2 and B1 from Hep-2 cells for stimulation; n, number of subjects with positive RSV antibody or INF-γ EliSpot test results; N, number of subjects tested; Min, minimum value; Max, maximum value.

a Median or average of antibody titer except for INF-γ EliSpot which is median spots/10^6^ cells.

b The cutoff value for a negative INF-γ EliSpot test result.

Next, we tested the plasma specimens from group A with the lysate EIA and the remaining group B, C, and D plasma specimens with both the lysate and F EIAs. We then combined group B and D results from the subset with these results (**Table 4**). All group A cord blood specimens were positive in the lysate EIA with a median titer of 89,281 (range of 19,903–2,776,302).

**Table 4:** Tests results for all specimens by group.

Assay	Group	*n*/*N* (%)	GMT (95% CI)	STD	Median^a^	Min	Max
INF-γ EliSpot	A	1/59 (2%)	5.1 (3.8, 6.9)	16.9	<25	<25	125
Lysate EIA	A	59/59 (100%)	105,033.7 (83,900.1, 131,490.7)	376,110.9	89,281	19,903	2,776,302
	B	39/39 (100%)	2,609.4 (1,933.7, 3,521.1)	3,115.3	2,449	271	11,312
	C	102/102 (100%)	5,693.5 (4,606.6, 7,036.7)	8,723.1	6291	415	45,081
	D	66/124 (53%)	4,909.4 (3,295.7, 7,313.1)	14,568.5	7,209^b^	202	65,614
F EIA	B	38/39 (97%)	3,510.9 (2,587.4, 4,763.9)	5,939.9	2,689	0	25,096
	C	101/102 (99%)	7,633.1 (5,895.3, 9,883.1)	16,949.2	8,798	0	81,484
	D	64/124 (52%)	8,658.8 (5,685.2, 13,187.6)	43,236.6	9,839^b^	209	228,917

Values for the INF-γ EliSpot are spots/10^6^ cord blood mononuclear cells.

Lysate EIA, IgG antibody enzyme immunoassay (EIA) with lysate from A2 and B1 infected Hep-2 cells antigen; F EIA, IgG antibody against expressed F protein antigen; INF-γ EliSpot, uses sucrose cushion purified RSV A2 and B1 from Hep-2 cells for stimulation; n, number positive; N, number tested; Min, minimum value; Max, maximum value.

a Median or average.

b Median group D specimens were calculated from 66 or 64 specimens with detectable antibody to focus this analysis on children likely to have been RSV-infected.

The results for all group C specimens supported the sub-study result of the high sensitivity of the lysate and F EIAs for detecting antibodies in children known to have been RSV-infected. Group C blood was collected at a median of 204 days, with a range of 184 to 300 days after enrollment (enrollment corresponds with the day RSV was detected). The lysate EIA detected antibody in 100% of the 102 group C specimens with a median titer of 6,291 (range, 415–45,081), and the F EIA detected antibody in 99% with a median titer of 8,798 (range, 0–81,484). The antibody titers were usually high with 75% having titers >2,819 with the lysate EIA and >3,227 with the F EIA. Importantly, the median age at blood collection was 302 days (range, 203–558). The group D specimens illustrate the use of these two assays to determine the immune status of children with unknown infection history. Lysate EIA antibody was detected in 53% with a median titer of antibody-positive specimens of 7,209 (range of 202–65,614) and F EIA antibody in 52% with a median titer of antibody-positive specimens of 9,839 (range, 209–228,917).

Group B specimens were collected as a second negative control for the EliSpot assay but, as noted, had an unexpectedly high rate of positivity. In a subset of 15 group B PBMCs, we detected a positive RSV EliSpot response in nine (60%). We assumed that the children in group B who were not exposed to an RSV season were not RSV-infected, and thus, the antibody titers provided information on the duration of maternal-acquired RSV antibodies. The 39 group B specimens were collected at a median of 138 days after delivery (range of 121 to 203 days), and all were positive with the lysate EIA and all but one with the F EIA with median titers of 2,449 (range of 271–11,312) and 2,689 (range of 0–25,096), respectively (**Table 4**). These antibodies are presumably maternal in origin and demonstrate that detectable antibodies are still present at ~4–6 months after delivery with these assays. In comparison, the group A cord blood specimens (antibody titer at delivery) had, as noted above, a lysate EIA median titer of 89,281 (range of 19,903–2,776,302). The calculated half-life of maternal-acquired antibodies was 54 days (**Supplementary Figure S1**). We did a second analysis in which we added the cord blood antibody titers to represent age day 0 (titer at birth) and calculated a half-life of 20 days.

## Discussion

We developed and characterized six antibody assays and one T-cell assay. All seven assays had acceptable performance including repeatability as indicated by CVs that were usually less than 30%, low level of reactivity against control antigen, and, in the case of the EIA antibody assays, minimal (between 6% and 12% median decrease in antibody titer) effect of red blood cell hemolysis, lipemia, or anticoagulation on antibody titer relative to a reference standard. We chose the fasting serum antibody titers as the reference standard for this comparison. Note that red blood cell hemolysis had a greater effect on the neutralization assay with a median decrease of 67% in antibody titer.

There were differences in the ability of these assays to detect evidence of RSV immunity in young children with two assays, the lysate and the F EIAs, being most sensitive. Both of these assays detected 44/44 (100%) of the subset group C specimens (children with an earlier RSV+ illness) used for the assay comparison. The other five assays, the subgroup A and subgroup B neutralizing antibody assays, the subgroup A and subgroup B IgG G protein antibody EIA assays, and the EliSpot assay, were positive in 86%, 95%, 43%, 57%, and 86%, respectively. The difference in the rate of detection was significantly higher for the lysate and F EIAs than that for the subgroup A neutralizing antibody assay, the Ga and Gb EIAs, and the EliSpot assay. These results suggested that either the lysate or F EIA detects maternal- or infection-induced antibodies in young children with high sensitivity. They also suggest that a combination assay is not likely to significantly improve detection with these assays. Consequently, we chose to use the lysate EIA to test the group A plasma specimens and both the lysate and F EIAs to test the remaining groups B, C, and D plasma specimens. The results for the remaining group C specimens supported our choice to focus on the lysate and F EIAs with 100% and 99% of the combined 102 group C specimens testing positive, respectively. The lysate and F EIA results for group D specimens suggested that ~50% of the children exposed to their first RSV season were infected. This infection rate is consistent with other studies (9–12, 36). Given the age at blood draw and antibody titer, it is possible that some of these antibody-positive specimens could be from maternal antibody and not induced by an earlier infection. Using a half-life of maternal antibody estimated in other studies of ~30 days (30, 54–57), the infant would have to have an initial titer, i.e., cord blood titer, >102,400 to still be positive (the cutoff for a positive titer is an estimated titer of ≥200) at 9 months of age and an initial titer >819,200 to still be positive at 12 months of age. In this study, the median age at blood draw for group D specimens was 390 days (range of 270 to 557 days), i.e., the infant’s age at blood draw was >9 months for all. Of the 26 specimens drawn between 9 and 12 months of age, six specimens had an estimated titer low enough (<1/1,200) to be from residual maternal antibody. Excluding these six specimens from the positive results would have a minimal effect on the estimate of RSV-infected children, i.e., a decrease from 53% to 48% for the lysate EIA and 52% to 47% for the F EIA. Conversely, antibodies induced by an intervening RSV infection will increase the estimates of the half-life of maternal antibody.

The high sensitivity of these antibody assays is consistent with that in a previously published study with the lysate EIA. In that study, the lysate EIA detected antibodies in 96% of 327 infants who had an earlier PCR-positive RSV infection (51). With the high sensitivity of the lysate and F EIAs in this study, there was little or no opportunity for the other assays to detect infections that were missed by the lysate and F EIA assays. Though these two assays detect RSV antibodies with good sensitivity; linking the detected antibody to prior infection in children less than 1 year of age is confounded by the presence of maternal antibodies. A combination of the antibody titer and the age at blood draw can sometimes suggest a likely source of antibody, i.e., with increasing age, the titer of the antibody that might be of maternal origin decreases. The usually high titer of infection-induced antibody supports this differentiation. Though less sensitive, the other assays provide information that may be relevant to some studies. Notably, the Ga and Gb EIAs are similar to the lysate and F EIAs in being relatively easy to perform while the neutralizing antibody assays and the EliSpot assays are technically more difficult.

Unexpectedly, 60% of 15 group B PBMC specimens were positive by the EliSpot assay. These PBMCs are from children unexposed to an RSV season, and very few, if any, were likely to have been infected with RSV. Thus, the rate of RSV-positive EliSpot responses is too high to be explained by off-season RSV infections and a positive response is not a specific RSV infection in young children. One explanation for the positive EliSpot responses is maternal microchimerism (58–61). A variety of cell types, including immune cells, are transferred from the mother to the fetus during pregnancy (maternal microchimerism), are present in the young infant, and can persist throughout life. The maternal cells can be detected in blood and various tissues including liver, spleen, and thymus. The role these cells play in fetal development, disease, and immunity to infection is under study. Maternal microchimerism could help protect the infant from RSV disease or sometimes contribute to disease. It is of interest that, in contrast to 60% of the infant specimens testing positive, only one of 59 group A cord blood specimens had a positive RSV ElisSpot test. This difference in positivity might reflect the expansion of maternal cells after delivery or post-delivery migration of immune cells resident in tissue into blood associated with infection or another stimulus. Others have noted variation in levels of maternal cells in infant blood specimens over time (61).

We estimated the decrease in maternally acquired antibodies over time, i.e., antibody half-life using group B specimens (from children unexposed to an RSV season and presumably RSV-naïve) alone and combined with group B specimens (cord blood). The group B specimens gave a half-life estimate of 54 days and the group B plus A specimens a half-life estimate of 20 days. These discrepant estimates are within the range of those previously reported (30, 54–57). The value of the maternal half-life estimates in this study, however, is limited by the lack of serial specimens from a child to directly determine antibody half-life and the relatively few group B specimens for a cross-sectional analysis.

In summary, these seven assays had acceptable performance in characterization studies, and studies with specimens from four carefully selected, prospectively enrolled groups with different types of RSV exposures demonstrate that two of the study assays, the lysate EIA and F EIA, were most sensitive for detecting RSV antibodies. Since in young children these antibodies can be maternal in origin or infection-induced, other information, e.g., age and antibody titer or IgA or IgM antibodies, is needed to determine if the child has had an RSV infection or not. The presence of IgA or IgM antibodies, which are not transferred efficiently from mother to infant, indicates infection as the source of antibodies (62, 63). The Lysate and F EIAs provide sensitive ways to detect RSV antibodies for clinical trials or epidemiologic studies.

## Data availability statement

The raw data supporting the conclusions of this article will be made available by the authors, without undue reservation.

## Ethics statement

The studies involving humans were approved by Institutional Review Board, Emory University School of Medicine. The studies were conducted in accordance with the local legislation and institutional requirements. Written informed consent for participation in this study was provided by the participant or the participants’ legal guardians/next of kin. Written informed consent was obtained from the minor(s)’ legal guardian/next of kin for the publication of any potentially identifiable images or data included in this article.

## Author contributions

LA: Conceptualization, Formal analysis, Funding acquisition, Methodology, Project administration, Resources, Supervision, Validation, Writing – original draft, Writing – review & editing. SJ: Data curation, Investigation, Methodology, Validation, Writing – review & editing. LH: Investigation, Project administration, Supervision, Writing – review & editing. BH: Conceptualization, Data curation, Investigation, Methodology, Validation, Writing – review & editing. CM: Formal analysis, Investigation, Methodology, Writing – review & editing. TG: Data curation, Investigation, Project administration, Supervision, Writing – review & editing. IY: Investigation, Methodology, Writing – review & editing. JY: Investigation, Methodology, Writing – review & editing. KS: Investigation, Methodology, Supervision, Writing – review & editing, Project administration. CKo: Investigation, Methodology, Writing – review & editing. CKa: Investigation, Methodology, Writing – review & editing. HS: Investigation, Methodology, Writing – review & editing. CL: Conceptualization, Methodology, Writing – review & editing. AJ: Writing – review & editing, Data curation, Formal analysis, Validation. CR: Writing – review & editing, Investigation, Methodology, Supervision. EA: Investigation, Methodology, Supervision, Writing – review & editing, Conceptualization, Funding acquisition, Project administration, Resources.
