# Supplementary material for: Development and comparison of immunologic assays to detect primary RSV infections in infants
Source: Front Immunol. 2024 Jan 12;14:1332772. doi: 10.3389/fimmu.2023.1332772 (PMC10811012; doi:10.3389/fimmu.2023.1332772)
Supplement: Supplementary file 1 [file DataSheet_1.doc]

**Table S1 Supplement.** Antibody titers by assay for a BEI high titer RSV serum

| Assay* | Number Tests | Median Titer |  | CV |
| --- | --- | --- | --- | --- |
| Lysate EIA | 12 | 253,650 |  | 0.21 |
| F EIA | 6 | 328,663 |  | 0.07 |
| Ga EIA | 6 | 54,074 |  | 0.13 |
| Gb EIA | 6 | 71,728 |  | 0.13 |
| A Neut | 6 | 1810 |  | 0.58 |
| B Neut | 6 | 1290 |  | 0.45 |

* A neut = Neutralizing antibodies against the subgroup A RSV strain A2; B neut = Neutralizing antibodies against the subgroup B RSV strain B1; Lysate EIA = IgG antibody enzyme immunoassay (EIA) with lysate from A2 and B1 infected Hep-2 cells antigen; F EIA = IgG antibody against expressed F protein antigen; Ga EIA = IgG antibody against expressed A2 strain G protein as antigen; Gb EIA= IgG antibody against expressed B1 strain G protein antigen.

**Table S2 Supplement.** Reactivity of the antibody EIAs against control antigen or negative control serum

|  | Hep-2 Control | | IgG Depleted Serum | |
| --- | --- | --- | --- | --- |
| Assay* | Average** | Range | Average | Range |
| Lysate EIA | 0.17 | 0.071-0.329 | 0.071 | 0.058-0.100 |
| F EIA | 0.137 | 0.045-0.268 | 0.061 | 0.036-0.110 |
| Ga EIA | 0.111 | 0.058-0.213 | 0.008 | -0.002-0.020 |
| Gb EIA | 0.101 | 0.045-0.197 | 0.005 | -0.002-0.015 |

*Lysate EIA = IgG antibody enzyme immunoassay (EIA) with lysate from A2 and B1 infected Hep-2 cells antigen; F EIA = IgG antibody against expressed F protein antigen; Ga EIA = IgG antibody against expressed A2 strain G protein as antigen; Gb EIA= IgG antibody against expressed B1 strain G protein antigen

**Each specimen test includes three wells with Hep-2 control antigen (N) and three wells with the respective RSV antigen (P). The average of P minus the average of N absorbance values is used to determine positivity and estimate titer. The IgG depleted serum is included on each plate and its P-N value is as part of the cutoff for a positive absorbance signal.

**Table S3 Supplement.** Effect of specimen quality or processing on results

| Assay* | Lipemia** | Red cell lysis | Anticoagulation |
| --- | --- | --- | --- |
| Lysate EIA | 1.06 | 1.17 | 1.31 |
| F EIA | 1.01 | 1.02 | 0.99 |
| Ga EIA | 1.06 | 1.16 | 1.1 |
| Gb EIA | 1.10 | 1.13 | 1.12 |
| A Neut | 0.99 | 1.67 | 1.06 |

* A neut = Neutralizing antibodies against the subgroup A RSV strain A2; Lysate EIA = IgG antibody enzyme immunoassay (EIA) with lysate from A2 and B1 infected Hep-2 cells antigen; F EIA = IgG antibody against expressed F protein antigen; Ga EIA = IgG antibody against expressed A2 strain G protein as antigen; Gb EIA= IgG antibody against expressed B1 strain G protein antigen.

**Data are the average ratio of the titer of fasting serum over titer of lipemic serum (collected after eating), hemolytic serum (fasting serum stored overnight at 4^o^C), or plasma (collected with citrate phosphate anticoagulant) for 5 specimens from adults. The various specimens were collected the same day.

Table S4. Significance of differences in assay positivity in Specimens from RSV+ Children (Subset of Group C)

| Comparison* | Assay | Fraction Positive n/N | Percent Positive (%) | p-value^a^ | BH Critical Value^b^ |
| --- | --- | --- | --- | --- | --- |
| A Neut vs Lysate EIA | A Neut | 37/44 | 84 | 0.012^#^ | 0.025 |
|  | Lysate EIA | 44/44 | 100 |  |  |
| B Neut vs Lysate EIA | B Neut | 42/44 | 95 | 0.494 | 0.042 |
|  | Lysate EIA | 44/44 | 100 |  |  |
| Ga EIA vs Lysate EIA | Ga EIA | 19/44 | 43 | <0.0001^#^ | 0.008 |
|  | Lysate EIA | 44/44 | 100 |  |  |
| Gb EIA vs Lysate EIA | Gb EIA | 21/44 | 48 | <0.0001^#^ | 0.017 |
|  | Lysate EIA | 44/44 | 100 |  |  |
| F EIA vs Lysate EIA | FEIA | 44/44 | 100 | NE | NE |
|  | Lysate EIA | 44/44 | 100 |  |  |
| ELISspot vs Lysate EIA | ELISpot | 38/44 | 86 | 0.026^#^ | 0.033 |
|  | Lysate EIA | 44/44 | 100 |  |  |
| Notes: Results displayed in this table are from a subset of 44 subject timepoints in Group C for which all assays were run. n=Number of subjects with RSV positive results. N=Number of subjects with available data. NE=Not estimable. Group C=Positive.  * A neut = Neutralizing antibodies against the subgroup A RSV strain A2; B neut = Neutralizing antibodies against the subgroup B RSV strain B1; Lysate EIA = IgG antibody enzyme immunoassay (EIA) with lysate from A2 and B1 infected Hep-2 cells antigen; F EIA = IgG antibody against expressed F protein antigen; Ga EIA = IgG antibody against expressed A2 strain G protein as antigen; Gb EIA= IgG antibody against expressed B1 strain G protein antigen; EliSpot= INF-γ EliSpot that uses sucrose cushion purified RSV A2 and B1 grown in Hep-2 cells to stimulate cells and values are spots/10^6^ peripheral blood mononuclear cells. ^a^ P-value calculated using Fisher's Exact test. ^b^ Critical value obtained from the Benjamini-Hochberg procedure. Significant p-values after application of BH procedure are noted by ^#^. | | | | | |

Figure S1. RSV antibody titers versus age in young children
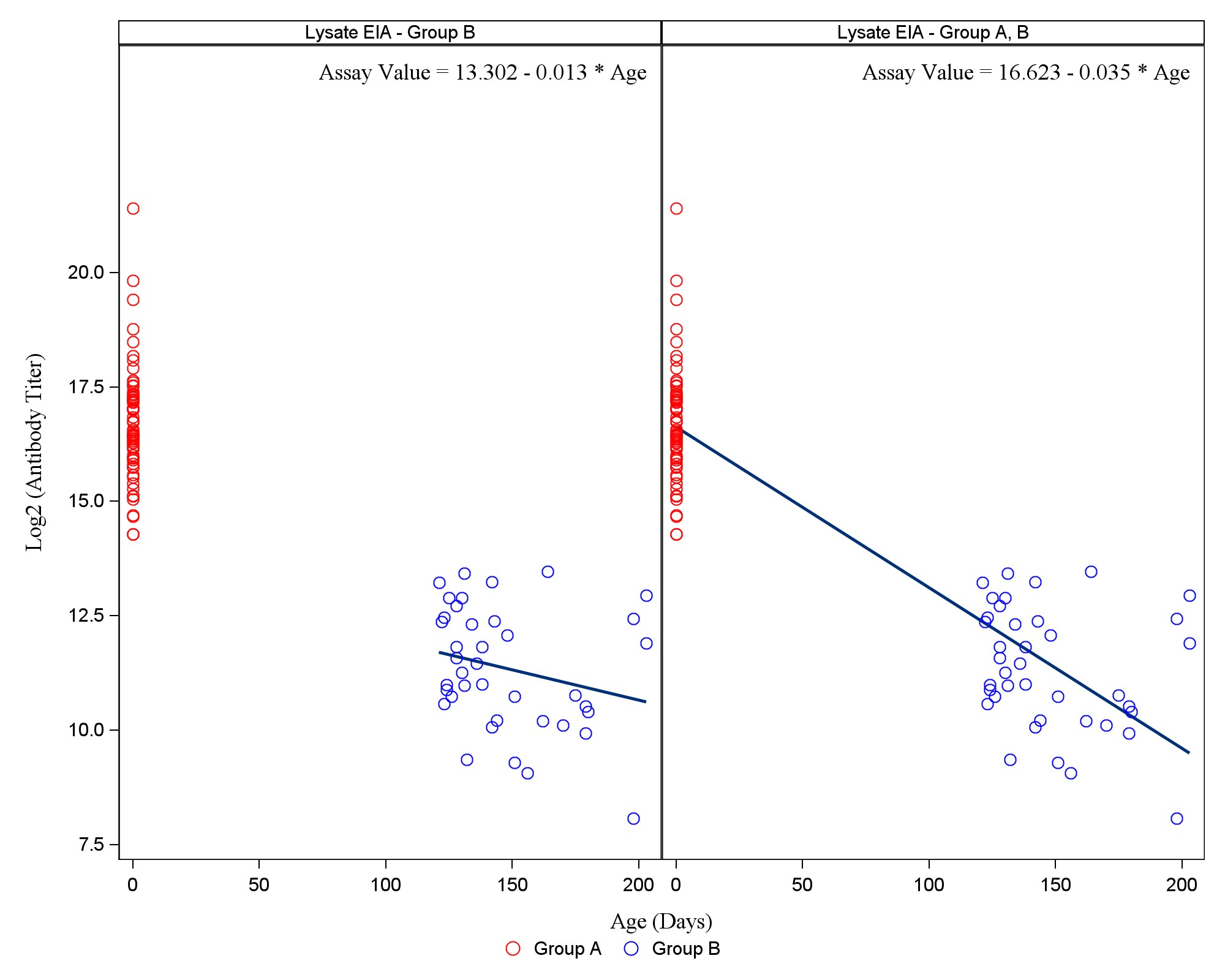


**Figure S1.** The antibody titer is estimated from absorbance values for the lysate EIA with RSV tissue culture lysate antigen. The specimens include 59 plasma samples from cord blood specimens (Group A) and 39 plasma specimens from infants not exposed to an RSV season (Group B).
